# TMP21 in Alzheimer’s Disease: Molecular Mechanisms and a Potential Target

**DOI:** 10.3389/fncel.2019.00328

**Published:** 2019-07-17

**Authors:** Kaixin Qiu, Xiaojie Zhang, Shuai Wang, Chunyan Li, Xin Wang, Xuezhi Li, Yili Wu

**Affiliations:** ^1^Cheeloo College of Medicine, Shandong University, Jinan, China; ^2^Shandong Collaborative Innovation Center for Diagnosis, Treatment and Behavioral Interventions, Institute of Mental Health, Jining Medical University, Jining, China; ^3^Shandong Key Laboratory of Behavioral Medicine, School of Mental Health, Jining Medical University, Jining, China; ^4^Department of Psychiatry, The Second Xiangya Hospital, Central South University, Changsha, China; ^5^National Clinical Research Center for Mental Disorders, Changsha, China; ^6^National Technology Institute on Mental Disorders, Changsha, China

**Keywords:** Alzheimer’s disease, TMP21, Aβ, Tau phosphorylation, neuronal loss

## Abstract

Alzheimer’s disease (AD) is the most common form of dementia in the elderly, which is characterized by progressive cognitive impairment. Neuritic plaques, neurofibrillary tangles and neuronal loss are the major neuropathological hallmarks in AD brains. TMP21 is a key molecule for protein trafficking in cells. Growing evidence indicates that TMP21 is dysregulated in AD, which plays a pivotal role in neuritic plaque formation. Therefore, we aim to review the dysregulation of TMP21 in AD, the role of TMP21 in neuritic plaque formation and underlying mechanisms. Moreover, the potential role of TMP21 in neurofibrillary tangle formation, synaptic impairment and neuronal loss is discussed. It will provide an outlook into the potential of regulating TMP21 as a therapeutic approach for AD treatment.

## Introduction

Alzheimer’s disease (AD) is the most common form of neurodegenerative disorders leading to dementia in the elderly. Neuritic plaques, neurofibrillary tangles and neuronal loss are the major neuropathological in the brain of patients with AD (Wu et al., 2014b; Wang et al., 2017). Clinical manifestations of AD are characterized by progressive memory loss, cognitive impairment and psychosis (Walsh and Selkoe, 2004; Sorbi et al., 2012). With the aggravation of aging population, the incidence of AD is increasing year by year. According to World Alzheimer Report 2018, 50 million people worldwide are living with dementia in 2018, and the number will be tripled to more than 152 million by 2050 (Patterson, 2018). The total estimated worldwide cost of dementia is US$1 trillion in 2018, and it will rise to 2 trillion dollars by 2030. Therefore, it is urgent to elucidate the pathogenesis of AD and develop effective treatments.

Growing evidence indicates that transmembrane protein, 21KD (TMP21), also known as transmembrane emp24 domain-containing protein 10 (TMED10), is a member of p24 family. It is dysregulated in AD and play a pivotal role in the pathogenesis of AD (Chen et al., 2006; Pardossi-Piquard et al., 2009; Zhang et al., 2019). Therefore, this review aims to describe the dysregulation of TMP21 in AD and its role in the pathogenesis of AD. Moreover, the underlying mechanismsare discussed. Furthermore, we provide an outlook into the potential of regulating TMP21 as a therapeutic approach for AD treatment.

## Characteristics of TMP21

### p24 Family Proteins

p24 family proteins are type I transmembrane proteins with molecular weight of 22–24 KD (Stamnes et al., 1995). Based on sequence homology, p24 proteins are classified into four subfamilies, i.e., α, β, γ, and δ (Dominguez et al., 1998; Strating et al., 2009; Pastor-Cantizano et al., 2016). In human, α subfamily consists of TMED9 and TMED11, and β subfamily consists of TMED2 and TMED4. γ family is the largest subfamily of p24 family, including TMED1, TMED3, TMED5, TMED6, and TMED7, while TMP21 belongs to δ subfamily. They share the conserved architecture, including luminal N-terminus, transmembrane (TM) region and cytosolic C-terminus. Generally, the luminal region contains a Golgi dynamic (GOLD) domain and a coiled-coil (CC) domain, while the cytosolic tail contains dilysine or diabasic (KKXX) motif and diaromatic (FF) motif. GOLD domain acts as a cargo receptor by mediating diverse protein-protein interactions, while CC domain mainly contributes to the oligomerization of p24 family members although a recent study showed that it does play a key role in recognition and transport of GPI-anchored proteins (Theiler et al., 2014). Oligomerization of p24 family members may affect their stabilization, localization, and expression levels (Carney and Bowen, 2004). For example, co-expression of TMED2 and TMP21 is necessary and sufficient for *cis*-Golgi/Golgi localization of each protein, while deleting each member individually leads to a general decrease of the other protein and loss function of the complex (Emery et al., 2000; Jenne et al., 2002). The cytosolic tail of p24 proteins contains signals for the binding of coat protein complex I (COPII) and coat protein complex I (COPI) contributing to the transport of cargo proteins between endoplasmic reticulum (ER) and Golgi apparatus in the early secretory pathway (Schimmöller et al., 1995; Muñiz et al., 2000; Barr et al., 2001; Belden and Barlowe, 2001; Gommel et al., 2001).

### Regulation of TMP21 Expression

Human TMP21 gene is located on chromosome 14q24.3, including 5 exons and 4 introns (Liu et al., 2011; Zhang et al., 2018), which is transcribed into two transcripts, Tmp21-I and Tmp21-II. With a nonsense mutation and a reading frame jump in comparison to Tmp21-I, Tmp21II was demonstrated to be derived from a pseudogene as the consequence of a duplication and diversification of hum-Tmp21-I. Thus, only Tmp21-I encodes a functional TMP21 (Blum et al., 1996; Horer et al., 1999). The expression of TMP21 is regulated at multiple levels. First, TMP21 is positively regulated by nuclear factor of activated T-cells (NFAT) at transcriptional level. In addition, sequence analysis showed that a number of putative *cis*-acting elements, e.g., CREB, YY1F, AP1 etc., are located in TMP21 promoter region, suggesting that TMP21 might be regulated by multiple transcriptional factors (Liu et al., 2011). Moreover, TMP21 expression could be regulated at post-transcriptional level. For example, the single-nucleotide polymorphism (SNP) rs12435391 in intron 4 significantly increases TMP21 expression by increasing the splicing efficiency of *TMP21* pre-mRNA (Zhang et al., 2018). Furthermore, TMP21is degraded through ubiquitin-proteasome pathway with a short half-life of approximately 3hs, indicating that TMP21 could be regulated at post-translational level (Liu et al., 2008).

### Tissue-Specific Expression and Functions *in vivo*

TMP21 is ubiquitously expressed in different tissues in mammalians. It was reported that TMP21 transcript is expressed in brain, pancreas, lung, liver, spleen etc. multiple organs of rats (Blum et al., 1996; Hosaka et al., 2007; Vetrivel et al., 2008). In human, both TMP21 mRNA and protein are highly expressed in heart, liver, spleen etc., whereas they are moderately expressed in brain, pancreas, colon etc. (Xie et al., 2014). In addition, TMP21 is expressed in most regions of the brain including septum, striatum, cortex, hippocampus, amygdala, thalamus, hypothalamus, cerebellum, and brainstem (Vetrivel et al., 2008). It shows stronger expression in neuronal cells than in glial cells (Vetrivel et al., 2008). Importantly, temporal expression of TMP21 was observed in mouse brain (Vetrivel et al., 2008). High level of TMP21 was detected in embryonic mouse brain, however, the expression of TMP21 gradually declined in brains of postnatal mice and reached lower level in adult brains. The evidence suggests that TMP21 expression is stringently regulated, which might play a pivotal role in development and maintenance of physiological functions. Consistently, complete deletion of TMP21 results in embryonic lethality at very early stage, while transgenic mice with neuron-specific increase of TMP21 expression display post-natal growth retardation and severe neurological problems including tremors, seizure, ataxia, uncoordinated movements and premature death (Denzel et al., 2000; Gong et al., 2011).

### Subcellular Distribution and Molecular Functions

TMP21 is a protein with 219 amino acids, including a signal peptide with 31 amino acids and a mature peptide with 188 amino acids. The signal peptide directs the newly synthesized TMP21 translocating into the ER, where it is cleaved and the mature TMP21 is generated. The mature TMP21 consists of N-terminal luminal region (32–185 aa), transmembrane region (186–206 aa) and C-terminal cytoplasmic region (207–219 aa), while the GOLD domain (41–193 aa) contains part of luminal region and part of transmembrane region, respectively (Anantharaman and Aravind, 2002; Nagae et al., 2016). TMP21 is enriched in the membrane of ER, plasma and Golgi apparatus. In addition, it was also reported to be located in ER-Golgi intermediate compartment (ERGIC) membrane and secretory vesicle membrane (Emery et al., 2000; Hosaka et al., 2007; Langhans et al., 2008; Strating and Martens, 2009). As a vesicle trafficking protein, the main function of TMP21 is for protein transport. First, TMP21 serves as a cargo receptor protein contributing to uptaking cargo proteins into COP vesicles, which is mainly mediated by GOLD domain (Muñiz et al., 2000; Anantharaman and Aravind, 2002; Castillon et al., 2009). The cytoplasmic tail of TMP21 is responsible for its binding to the subunits of COPI or COPII contributing to the transport of cargo proteins between ER and Golgi apparatus (Muñiz et al., 2000; Belden and Barlowe, 2001; Gommel et al., 2001; Blum and Lepier, 2008). Secondly, TMP21 cytoplasmic tail combined with the Golgi matrix proteins has an effect on the transport of cargo proteins to the cell surface or the efficient retention of them in the Golgi apparatus (Barr et al., 2001; Blum and Lepier, 2008). In addition, TMP21 also selectively interacts with glycosylphosphatidylinositol-anchored proteins contributing to their ER export and lipid rafts translocation. Moreover, TMP21 is a major component of Golgi apparatus and *cis*-Golgi network (CGN), which is essential for the integrity and proper organization of Golgi structure. Furthermore, TMP21 is a modulator of γ-secretase, which is implicated in various physiological and pathological processes by cleaving its substrates, e.g., APP and Notch. γ-secretase is a protein complex consisting of the core catalytic components, presenilin1 (PSEN1) and presenilin2 (PSEN2), and the regulatory components, nicastrin, APH-1 and PEN-2. TMP21 is a non-essential component of the complex (Pardossi-Piquard et al., 2009; Wang et al., 2017). Importantly, TMP21 regulates γ-secretase cleavage at the γ-site but not at the ε-site, indicating that TMP21 selectively regulates γ-secretase cleavage on its substrate. For example, TMP21 regulates γ-secretase cleavage of APP at the γ-site, however, it has no effect on the γ-secretase cleavage of Notch at the ε-site (Chen et al., 2006; Bromley-Brits and Song, 2012). Compared with other p24 members, it is a specific feature of TMP21 to regulate γ-secretase activity.

## Aberrant Expression of TMP21 in AD

Increased evidence indicates that TMP21 is dysregulated in AD. First, Chr14q24.3 is defined as a minimal co-segragating region by linkage studies, which contains the major genes predisposing to early onset AD. *TMP21* gene is located in this region. Secondly, the expression of TMP21 are significantly reduced in brains of both sporadic AD cases and familial AD cases compared with age-matched controls, which is consistent with the previous study that knockdown of TMP21 increases Aβ expression (Chen et al., 2006; Vetrivel et al., 2008). In addition, the SNP rs12435391 in intron 4 of *TMP21* gene is associated with increased AD risk by accelerating *TMP21* pre-mRNA splicing leading to increased expression of TMP21 (Zhang et al., 2018). It seems contradictory that both reduced and increased TMP21 levels are associated with AD. However, two issues need to be considered. First, it has to be noted that the latter experiment was done in HEK293 cells but not in neurons. The exact role of SNP rs12435391 in the regulation of TMP21 expression in neuronal cells or brains needs to be further investigated. Secondly, the precise control of TMP21 expression is crucial to maintain the physiological functions as both increased and decreased TMP21 expression is lethal in mice (Denzel et al., 2000; Gong et al., 2012). It suggests that TMP21 might has bidirectional roles, which is common for many important molecules, such as regulator of calcineurin 1 (Wu and Song, 2013; Yan et al., 2014; Duan et al., 2015; Wu et al., 2015). Thus, it highly indicates that the precise control of TMP21 expression is crucial to maintain its physiological functions, avoiding the pathogenic effects.

Dysregulated calcineurin-NFAT signaling might be implicated in the dysregulation of TMP21 in AD as TMP21 is positively regulated by NFAT at transcriptional level (Abdul et al., 2009; Liu et al., 2011; Sun et al., 2014; Wu et al., 2014a). First, the regulator of calcineurin 1 (RCAN1) is significantly increased in AD brains, which inhibits calcineurin-NFAT signaling (Wu and Song, 2013). It suggests that increased RCAN1 may contribute to the reduction of TMP21 expression in AD. In addition, elevated oligomeric Aβ stimulates calcineurin-NFAT signaling in neurons and astrocytes, which might promote TMP21 expression in AD (Shankar et al., 2007; Abdul et al., 2009). Moreover, a number of putative *cis*-acting elements located in TMP21 promoter region might be implicated in the dysregulation of TMP21 transcription as various transcriptional factors are altered in AD, such as CREB (Liu et al., 2011; Pugazhenthi et al., 2011; Bartolotti et al., 2016; Ettcheto et al., 2018). Furthermore, impairment of ubiquitin-proteasome system in AD might contribute to TMP21 upregulation as TMP21 is degraded through ubiquitin-proteasome pathway (Liu et al., 2008). Therefore, aberrant expression TMP21 in AD is a combined effect by various regulation mechanisms, and differential alteration of TMP21 observed in AD might be associated with the progress or stages of AD.

## TMP21 in the Pathogenesis of AD

Neuritic plaques, neurofibrillary tangles and neuronal loss are the characteristic neuropathological features in AD brains (Wu et al., 2014a,b; Wang et al., 2017). Increased evidence indicates that dysregulated TMP21 promotes neuritic plaque formation. Moreover, TMP21 dysregulation potentially contributes to neurofibrillary tangle formation, synaptic impairment and neuronal loss.

### Aberrant Expression of TMP21 Promotes Aβ Generation

Neuritic plaques are only observed in AD while neurofibrillar tangles, synaptic impairment and neuronal loss also exist in many other neurodegenerative disorders. Thus, neuritic plaques are the only neuropathological hallmark to distinguish AD from other neurodegenerative disorders. Amyloid β-protein (Aβ) is the major component of neuritic plaques, which is derived from sequential cleavages of APP by β- and γ-secretase. However, the majority of APP undergoes non-amyloidogenic pathway. First, APP is cleaved by α-secretase to generate a C-terminal fragment (CTF) of 83 amino acids (C83), excluding Aβ generation (Wang et al., 2017). In addition, beta-site APP cleaving enzyme 2 (BACE2) and η-secretase are also involved in non-amyloidogenic pathway although our recent work showed that BACE2 is a conditional β-secretase under specific conditions (Willem et al., 2015; Wang et al., 2019). The minority of APP is cleaved by beta-site APP cleaving enzyme 1(BACE1) at Asp1 (β site) and Glu11 (β′ site, numbering for Aβ) sites, respectively. Glu11 is the major β-cleavage site to yield a CTF with 89 amino acids (C89). C89 is further cleaved by γ-secretase to produce a truncated Aβ_11-40/42_, excluding Aβ generation. Asp1 is the minor β-cleavage site to generate a CTF with 99 amino acids (C99) which is further cleaved by γ-secretase to produce Aβ (Liu et al., 2002; Deng et al., 2013).

**Figure 1:**
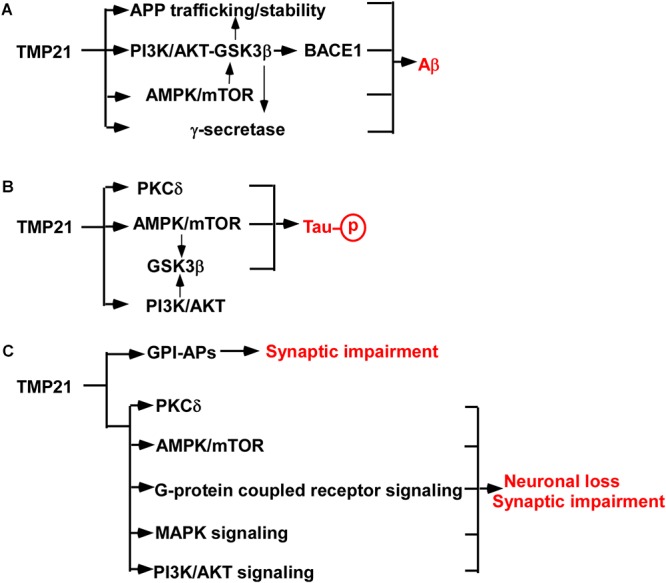
TMP21 in the pathogenesis of AD. **(A)** TMP21 dysregulation promotes Aβ generation. **(B)** TMP21 dysregulation might increase Tau phosphorylation. **(C)** TMP21 dysregulation might contribute to synaptic impairment and neuronal loss.

Increased evidence indicates that dysregulated TMP21 is implicated in increased Aβ generation in AD (Figure 1A). First, γ-secretase is a protein complex consisting of PSEN1 or PSEN2, nicastrin, APH-1 and PEN-2, while TMP21 is a non-essential component of the complex (Wang et al., 2017). As a modulator of γ-secretase, reduced TMP21 expression promotes Aβ generation by increasing γ-secretase cleavage of APP at γ-site (Chen et al., 2006; Bromley-Brits and Song, 2012). Secondly, TMP21, likely other p24 family proteins, regulates the trafficking of APP, which further affects Aβ production. For example, knockdown of TMP21 expression by specific siRNA increases the stability and maturation of nascent APP in both non-neuronal and neuroblastoma cell lines, contributing to increased APP level and Aβ generation. Moreover, TMP21 suppression compromises bidirectional transport of APP in the ER/Golgi, resulting in more APP undergoing amyloidogenic cleavage in endocytic compartments, as well as secretion of sAPP, Aβ40 and Aβ42 (Vetrivel et al., 2007). Our recent work showed that reduced TMP21 inhibits the expression of phosphoinositide-3-kinase regulatory subunit 1(PiK3r1) in mouse brains, which might release the inhibitory effect of PI3K/AKT on GSK3β contributing to increased activity of GSK3β (Zhang et al., 2019). Increased GSK3β activity promotes APP phosphorylation facilitating APP stability and Aβ generation (Wang et al., 2017). On the other hand, increased GSK3β activity felicitates NF-κB-mediated BACE1 expression and activity, which further promotes APP processing and Aβ generation (Ly et al., 2013).

AD-associated SNP rs12435391 in intron 4 of *TMP21* gene increases TMP21 expression, which promotes Aβ generation (Zhang et al., 2018). It remains unknown that this effect is mediated by regulating γ-secretase activity or modification/trafficking of APP or BACE1 activity. In addition, as a vesicle trafficking protein, aberrant expression of TMP21 might also regulate the trafficking and stability of multiple proteins involved in APP processing (e.g., APP, α-secretase, BACE1, BACE2), leading to increased Aβ generation. For example, TMP21 does affect APP stability contributing to the alteration of Aβ production (Vetrivel et al., 2007). Furthermore, TMP21 might be implicated in mTOR- or PI3K-mediated GSK3β activation, which subsequently contributes to increased Aβ generation by upregulating APP expression, BACE1 expression and γ-secretase activity (Qing et al., 2008; Caccamo et al., 2013; Ly et al., 2013; Yang et al., 2013; Xu et al., 2015; Wang et al., 2017; Zhang et al., 2019). However, it has to be noted that the effect of increased TMP21 on promoting Aβ generation was determined in HEK293 cells but not in neurons. The exact role of SNP rs12435391 in Aβ generation in brain or neuronal cells needs to be further investigated.

TMP21 possibly plays a bidirectional role in Aβ production as both upregulation and downregulation of TMP21 promotes Aβ generation in HEK293. In addition, TMP21 dysregulation does show bidirectional functions in mice as both the increase and decrease of TMP21 expression is lethal in mice (Denzel et al., 2000; Gong et al., 2012). Moreover, the bidirectional roles are common for many important molecules. For example, both upregulation and downregulation of regulator of calcineurin 1 facilitates neuronal apoptosis (Wu and Song, 2013; Yan et al., 2014; Duan et al., 2015; Wu et al., 2015). Thus, the effect of TMP21 dysregultion on Aβ generation in neuronal cells and underlying mechanisms need to be further investigated.

### Potential Roles of Dysregulated TMP21 in Tau Phosphorylation and Neuronal Loss

The major component of neurofibrillary tangles is hyperphosphorylated Tau. Increased Tau phosphorylation promotes neurofibrillary tangle formation (Wu et al., 2014b). Although no direct evidence supports that TMP21 plays a role in Tau phosphorylation, a couple of studies indicates that TMP21 might contribute to the regulation of Tau phosphorylation (Figure 1B) (Tsujio et al., 2000; Wang et al., 2011; Caccamo et al., 2013; Xu et al., 2015; Lee et al., 2017; Zhang et al., 2019). First, increased Aβ promotes Tau hyperphosphorylation via various mechanisms, e.g., oxidative stress, while TMP21 dysregulation contributes to Aβ generation (Wu et al., 2014b). It suggests that dysregulated TMP21 might promote Tau hyperphosphorylation mediated by increasing Aβ generation. In addition, increasing mTOR activity promotes Tau phosphorylation, while reduced TMP21 expression facilitates the activation of mTOR (Caccamo et al., 2013; Xu et al., 2015). It suggests that reduced TMP21 might promote mTOR-mediated Tau phosphorylation contributing to neurofibrillary tangle formation in AD (Lee et al., 2017). Moreover, protein kinase C delta (PKCδ) inactivates GSK3β-mediated Tau phosphorylation, while TMP21 inhibits PKCδ activation (Tsujio et al., 2000; Wang et al., 2011). Consistently, our recent work indicates that reduced TMP21 might increase GSK3β activity by downregulating PiK3r1 expression (Zhang et al., 2019). It suggests that aberrant expression TMP21 might play a role in Tau hyperphosphorylation contributing to neurofibrillary tangle formation in AD via various mechanisms.

Synaptic impairment and neuronal loss is the major cause leading to cognitive impairment and psychosis in AD. However, the role of aberrant expression of TMP21 in synaptic dysfunction and neuronal loss is elusive. Growing evidence indicates that TMP21 might be involved in synaptic dysfunction and neuronal loss in AD (Figure 1C). First, changes in TMP21 expression might be implicated in the dysfunction of ER processing and export of glycosylphosphatidylinositols-associated proteins (GPI-APs), which acts as a modulator of synapse development via direct interactions with key synapse-organizing proteins (Um and Ko, 2017; Kim et al., 2019). TMP21 is a member of p24 protein which specifically recognizes GPI-APs and is essential for GPI-APs quality control. Either dysregulation or dysfunction of TMP21 may lead to abnormal synaptic stability via faulty quality-control of GPI-APs, which has been reported in the frontal cortex of elderly subjects with schizophrenia (Kim et al., 2019). Consistently, TMP21 reduction significantly affects synapse-associated biological processes and pathways in mouse brains, e.g., synaptic development, synapse organization and synaptic vesicle cycle etc. (Zhang et al., 2019). Moreover, TMP21 inhibits mTOR- and PKCδ-mediated apoptosis in cancer cells, while dysfunction of mTOR- and PKCδ is implicated in synaptic impairment and neuronal apoptosis (Caccamo et al., 2010; Wang et al., 2011; Xu et al., 2015). It suggests that dysregulation of TMP21 might be implicated in synaptic impairment and neuronal apoptosis. In addition, TMP21 reduction could lead to the dysregulation of multiple pathways, e.g., G-protein coupled receptor signaling pathway, MAPK signaling pathway, PI3K-AKT pathway etc., which play an important role in synaptic impairment and neuronal apoptosis, such as apelin and apelin receptor system (Franco et al., 2017; Qiu et al., 2017; Wu et al., 2017, 2018; Zhang et al., 2019). Furthermore, TMP21 dysregulation facilitates Aβ generation and potentially increases Tau hyperphosphorylation, which subsequently promotes synaptic impairment and neuronal loss. For example, Aβ-induced dysregulation of an NMDA-type glutamate receptor-dependent signaling promotes synapse loss, while TMP21 dysregulation promotes Aβ generation. It suggests that dysregulated TMP21 might trigger synapse loss through Aβ-induced dysfunction of the NMDA-type glutamate receptor (Shankar et al., 2007). Hyperphosphorylation of Tau promotes neuronal apoptosis, while TMP21 potentially increases Tau phosphorylation, suggesting that dysregulated TMP21 might promotes neuronal apoptosis by increasing Tau phosphorylation (Lee et al., 2017). Therefore, TMP21 dysregulation might contribute to synaptic impairment and neuronal loss, which is mediated by the direct effect of TMP21 dysregulation or the indirect effect of TMP21 dysregulation-induced increased Aβ generation and Tau phosphorylation.

## Conclusion

Growing evidence indicates that TMP21 might play a central role in the pathogenesis of AD and it might be a specific and effective target for AD treatment. First, dysregulated TMP21 promotes Aβ generation by modulating APP trafficking/stability and γ-secretase activity, while it potentially regulates BACE1 expression and activity. Importantly, TMP21 regulates γ-secretase cleavage of APP at the γ-site, however, it has no effect on the γ-secretase cleavage of Notch at the ε-site. It is well-known that inhibiting Notch cleavage and its functions is a major obstacle to develop γ-secretase inhibitors for AD treatment. The specific feature of TMP21, inhibiting γ-cleavage but sparing ε-cleavage, makes it as a potential target to reduce Aβ generation and avoid toxic effects. Although no direct evidence shows that TMP21 plays a role in Tau pathology and synaptic/neuronal loss, a number of studies suggest that TMP21 dysregulation might be implicated in Tau pathology and synaptic/neuronal loss. Above evidence indicates that modulating TMP21 expression is a potential target for AD treatment. However, targeting TMP21 for AD treatment is limited by the following reasons. First, developing an approach to precisely controlling TMP21 expression is crucial for clinical application. Moreover, elucidating a number of issues is critical for developing effective TMP21-targeting approaches, e.g., the consistence of TMP21 regulation in neurons and non-neuronal cells, the physiological function of TMP21, temporal regulation of TMP21 during AD progress etc.

## Author Contributions

YW conceived and formulated the manuscript. KQ, XZ, SW, CL, XW, and YW wrote the manuscript. XZ, XL, and YW revised the manuscript.

## Conflict of Interest Statement

The authors declare that the research was conducted in the absence of any commercial or financial relationships that could be construed as a potential conflict of interest.
